# Service coverage and health workforce allocation strategies for geriatric and palliative care in low- and middle-income countries: A protocol for a systematic review and meta-analysis

**DOI:** 10.1097/MD.0000000000029030

**Published:** 2022-03-11

**Authors:** Dipika Shankar Bhattacharyya, Md. Hasibul Hossain, Goutam Kumar Dutta, Iffat Nowrin, KM Saif-Ur-Rahman

**Affiliations:** Health Systems and Population Studies Division, icddr, b, Dhaka, Bangladesh.

**Keywords:** geriatric care, health workforce, human resources for health, LMICs, palliative care, service coverage

## Abstract

**Background:**

Advances in medical science coupled with increased people's income results an elevated average of life expectancy even in the resource poor countries. The growing number of aged population, however, has drawn little attention in health system discourse of low- and Middle-Income Countries (LMICs). Nevertheless, ensuring availability of appropriate service and properly trained and skilled health workforce is an absolute necessity for a functional geriatric and palliative healthcare. Given the lack of specialist geriatricians in LMICs contexts, there are other health workforce strategies that might be effective in building a proper health system response to this growing demand. Therefore, we aimed to identify and synthesize evidence on the existing health workforce-related strategies taken to provide geriatric and palliative care in LMICs.

**Methods:**

We will follow the recommendations provided by the Preferred Reporting Items for Systematic Reviews and Meta-Analyses (PRISMA) checklist. Following the PRISMA guidelines, we will search the Medline/PubMed, Scopus, Web of Science and Cochrane database from January 2011 to December 2021 using a comprehensive search strategy. Two independent reviewers will screen the title and abstracts text using the specified inclusion and exclusion criteria. For the finally included articles, full manuscripts will be retrieved, and reviewers will appraise and extract data using standardized form independently. The third reviewer will resolve any disagreements appear in the process. The findings of the review be synthesized using the narrative synthesis approach to analyse descriptive quantitative and qualitative data. Furthermore, meta-analysis will be done provided that the data meet certain requirement as per Cochrane guideline. Rayyan software will be used to manage and synthesize data. Revman software will be used to do meta-analysis, if data support.

**Results:**

Findings of this review will be published in a peer-reviewed journal.

**Conclusion:**

This systematic review will identify the existing effective strategies taken to provide geriatric and palliative care, in LMICs.

## 1 Introduction

The number of people aged 65 years or over has risen significantly in last few decades in many countries across the world.^[1,2]^ Evidence suggests, globally the number of people aged 65 years or over is expected to grow by 56%, from 901 million to 1.4 billion, within the period of 2015 to 2030.^[3]^ Besides, by 2050 the number of elderly people is expected to be double its size in 2015, reaching nearly 2.1 billion.^[3,4]^ A vast majority of this growing aged population will be placed across the low- and middle-income countries (LMICs).

However, the health system in these countries is yet to be aligned with this demographic transition of growing aged population.^[5,6]^ The rapid growth of the old-age population has spared little time for social and health systems to adapt.^[4]^ In fact, in the LMIC country context, care for aged population has never been the focus of much health system discourse due to the presence of other public health problems like maternal and child mortality. But, in old age, people have weakened functioning due to progressive loss of cognitive function and functional competency that results not only the onset of acute chronic diseases but also frailty.^[7]^ Previous studies and systematic reviews show that while the older people suffer from numerous ailments and diseases, the most common issues are cancers,^[8]^ diabetes,^[9]^ poor oral health issues,^[10]^ and dementia.^[11]^ Also, the old aged person needs geriatric mental health services^[12,13]^ which is yet to get proper attention from policy perspective in LMIC setting.

In recent years there are several international calls that recognizes the importance of geriatric and palliative care services across the world.^[14]^ However, despite this recognition, insufficient access to these services is disproportionally burdened among the people living in LMICs. Only 9% of the world's countries, all of which are relatively high income, are considered to have advanced levels of geriatric and palliative care integration into mainstream health services.^[15]^ There is little or no access to these services in majority of LMICs, and an estimated 20 million people have unmet care needs.^[16]^ While it is being recognized that unmet geriatric and palliative healthcare needs are prevalent among the aged people in LMIC, there is lack of evidence on the service coverage and kind of service package are now being provided in those countries.

These issues are further complicated by the recent evidence that suggests elderly people from the LMIC setting often are subjected to verbal and psychological abuse and face wide range of discrimination in the healthcare setting while seeking care.^[17,18]^ Factors associated with this maltreatment are lack of enough staff, inadequate training and qualification of existing staff, stress and burnout among staff, and inadequate legislative protection in the care of older people.^[19]^ It has been regarded that geriatric and palliative care entails a special body of knowledge and the workforce providing these services need to be specialized in the area.^[20,21]^ The workforce need to be trained enough to deliver the complex functional or medical service to the older adults with compassion.^[22]^ In the high-income country settings, the specialization in geriatrics has been evolved over the last century and several hospital and residential model has already been developed for ensuring effective services to the old aged population.^[23,24]^ On the other hand, there is inadequate knowledge from across the LMICs on how these services are provided.

Considering the global call for action on achieving Universal Health Coverage and Sustainable Development Goals, a crucial first step in ensuring the proper geriatric and palliative care services is to understand the existing service gap and workforce allocation strategies in LMIC setting. This might help policymakers to formulate context-specified intervention for ensuring these services. The proposed systematic review and meta-analysis aims to identify and synthesize available evidence on the service coverage and health workforce allocation strategies for providing geriatric and palliative care in LMICs.

## 2 Methods

This review will follow Preferred Reporting Items for Systematic Review and Meta-Analysis (PRISMA-P) guidelines. This review has also been registered in the International Prospective Register of Systematic Reviews (PROSPERO) with registration number CRD42021287982. The PRISMA-P checklist for systematic review protocol has been attached as a supplementary file. The study started on December 2021 and the estimated end date is May 2022.

### 2.1 Study design

We will include quantitative, qualitative, and mixed method studies. Besides we will include both primary observational and experimental studies for finding evidence on the service coverage and workforce for managing geriatric and palliative care in the LMICs context.

### 2.2 Information sources

We will search the following electronic databases to retrieve relevant articles: PubMed/MEDLINE, Web of Science, Scopus, and Cochrane database of systematic reviews. Besides, the search will also include the grey literature from the website and online repositories of relevant funding organizations like Swedish International Development Cooperation (SIDA), Canadian International Development Agency (CIDA), Centre for Communicable Disease Prevention and Control (CDC), WHO Library Database/The bibliographic database (WHOLIS), United States Agency for International Development (USAID), World Bank, Department for International Development (DFID), and United Nations Development Programme (UNDP).

### 2.3 Inclusion criteria

This review will use the PICO (Population, Intervention, Comparison, Outcomes) framework to set the inclusion criteria. The detail of the PICO is provided below.

### 2.4 Population

The review will include studies reporting on population aged 65 years of age and above. The study population will not be limited in terms of sex, place of residence (urban or rural), ethnic identity, and socioeconomic status. Studies that are conducted only in the LMICs, as defined by World Bank, will be included in the review.

### 2.5 Interventions

The proposed systematic review and meta-analysis will focus on the identifying strategies to provide geriatric and palliative care in LMICs. The interventions specifically focused on the strengthening of health workforce such as “geriatrician”, “physician”, “nurse”, will be considered for this review. Besides, the geriatric care interventions that are provided in hospitals, clinics, hospice, home-based care, old home, will be included for capturing information on types and contents of service for old aged people.

### 2.6 Comparator

As we will not evaluate the effectiveness of types of intervention, we will not use any comparator for this review.

### 2.7 Outcome

The main outcome of the study is the assessment of geriatric and palliative services available in the LMICs. The services that are provided by health system through hospitals, clinics, and residential care facilities will be included. All geriatric and palliative issues, for example, dementia, depression, anxiety, frailty of health, end of life-related emotional and spiritual support, relaxation techniques; will be considered from the review. The additional outcome will be the allotment of dedicated healthcare staff, doctors, nurses, and other relevant trained health care workers, including but not limited to allied healthcare staff, hygienists, personal care workers in geriatric and palliative care. Besides provision for a geriatric specialist, outsourcing to provide geriatric and palliative care, in LMICs will also be analyzed as an additional outcome. In addition, any incentive strategy for the geriatric workforce will also be reported as the additional outcomes.

### 2.8 Exclusion criteria

Studies that are not conducted in LMICs and published before the year 2011 will be excluded from the review. Besides, trial or review protocols, systematic review, conference papers, and book chapters will be considered for exclusion. Articles published in any other language apart from English will also be excluded.

### 2.9 Search strategy

A comprehensive search strategy will be developed for each of the electronic and bibliographic databases that include Medline/PubMed, Scopus, Web of Science, and the Cochrane Library. The key search terms for Population Intervention and Outcome has been demonstrated in Table 1. Using the subject heading terms and keyword we already have developed the search strategy for PubMed that has been shown in Table 2. For the other databases search strategy will be prepared according to the database-specific filters, as applicable. Moreover, additional references from the included articles will also be searched. Besides, we will contact the authors for obtaining relevant information and if full text is not available online.

**Table 1:** Key terms for population, intervention, and outcome.

Population (P)	Intervention (I)	Outcome (O)
“Aged”, “Older people”, 65 and over”, LMICs.	“Geriatrician”, “physician”, “nurse”, “geriatric nurse”, “trained health care workers” “health provider”’ “Hospital”, “clinics”, “hospice” “home-based care”, “old home”,	“human resource management”, “staff deployment”, “staff development”, “hospital administration”, “training”, “Geriatric health service”, “palliative care provision”, “psychosocial support”, “psychological support”, “rehabilitation support”, “emotional support,

LMICs = low- and middle-income countries.

**Table 2:** Search strategy for PubMed/Medline.

Search number	Search terms
#1	Aged[Title] OR aging[Title] OR elder[Title] OR elderly[Title] OR “frail elderly”[Title] OR Geriatric[Title] OR “Older adult”[Title] OR “Older patient”[Title] OR “Older people”[Title] OR “Older person”[Title] OR “older”[Title] OR Palliative[Title] OR pensioner[Title] OR “retired person”[Title] OR “senior citizen”[Title] OR Senior[Title] OR aged[Title] OR “65[Title] AND over”[Title] OR “over 65”[Title] OR “over sixty-five”[Title] OR “over sixty five”[Title]
#2	Geriatrician[Title] OR physician[Title] OR doctor[Title] OR specialists[Title] OR clinician[Title] OR consultant[Title] OR “medical personnel”[Title] OR nurse[Title] OR “nursing staff”[Title] OR “nursing workforce”[Title] OR “geriatric nurse”[Title] OR “specialist nurse”[Title] OR “healthcare worker”[Title] OR “medical staff,[Title] OR caregiver[Title] OR “trained health care workers”[Title] OR “health provider”[Title] OR Hospital[Title] OR clinics[Title] OR hospice[Title] OR “home-based care”[Title] OR “old home”[Title] OR “long term care facility”[Title] OR “home care service”[Title] OR “Home for the aged”[Title] OR “home visit”[Title] OR “house visit”[Title] OR “home-based facilities”[Title] OR “home-based nursing”[Title] OR “critical care”[Title] OR “outpatient facilities”[Title] OR “outpatient center”[Title] OR “residential facilities”[Title]
#3	“human resource management”[Title/Abstract] OR “health workforce management”[Title/Abstract] OR “work distribution”[Title/Abstract] OR “work organization”[Title/Abstract] OR “staff deployment”[Title/Abstract] OR “staff development”[Title/Abstract] OR “hospital administration [Title/Abstract] OR training[Title/Abstract] OR “skill management”[Title/Abstract] OR “skill-mix”[Title/Abstract] OR “skill-oriented staffing”[Title/Abstract] OR “Task shifting”[Title/Abstract] OR retention[Title/Abstract] OR treatment[Title/Abstract] OR “Geriatric health service”[Title/Abstract] OR “palliative care provision”[Title/Abstract] OR “geriatric long-term care”[Title/Abstract] OR “Long-term care”[Title/Abstract] OR “critical care”[Title/Abstract] OR “health management”[Title/Abstract] OR “social care”[Title/Abstract] OR “psychosocial support”[Title/Abstract] OR “psychological support”[Title/Abstract] OR “rehabilitation support”[Title/Abstract] OR “emotional support” [Title/Abstract]
#4	#1 AND #2 AND #3

### 2.10 Study selection

The selection of studies will be done in 2 stages. At the first stage, all the studies retrieved, after applying search strategy, will be uploaded into Rayyan – an open source web-based tool designed to screen articles for systematic review and knowledge synthesis. Once the articles are imported to Rayyan, we will identify and remove the duplicates. After that 2 reviewers will independently screen and select the articles applying the inclusion and exclusion criteria.

**Figure 1:**
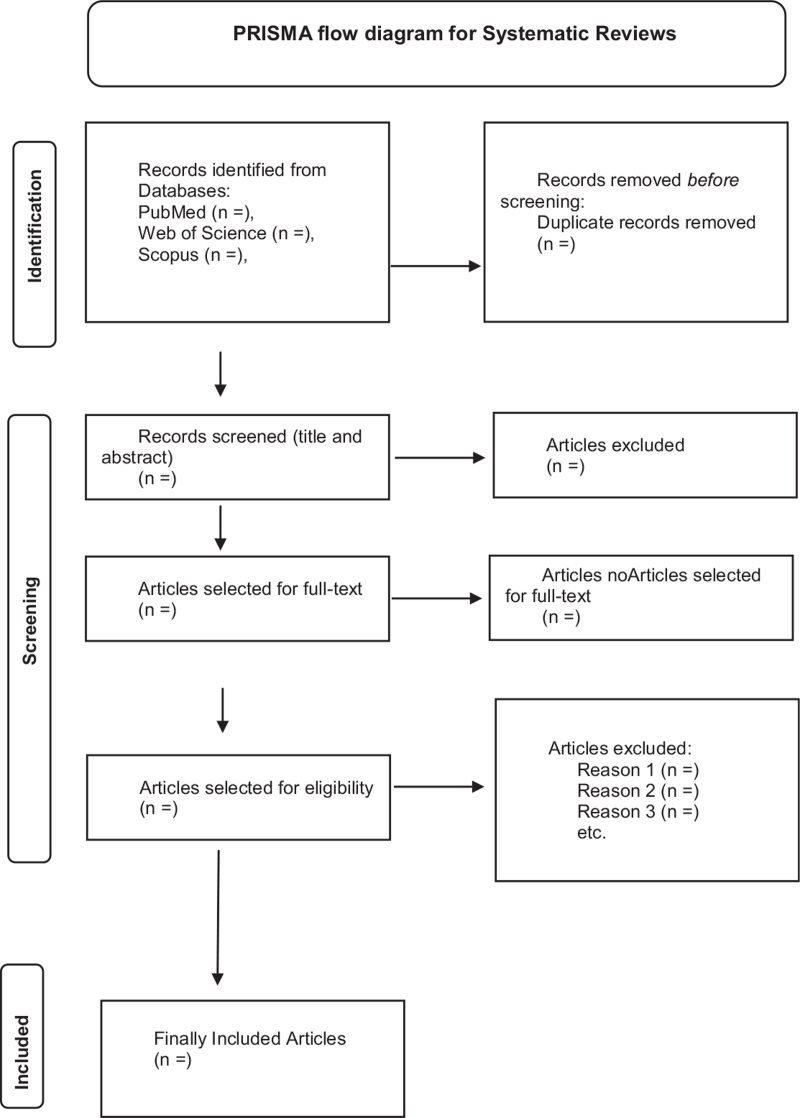
PRISMA flow diagram of systematic review.

In the second stage, we will retrieve the full text of the qualified studies. Afterward, the 2 reviewers will independently screen the full text according to the selection criteria. For exclusion of each articles the reviewers will take note of the reasons which will be reported later. Any disagreements or discrepancies appearing in the screening process will be resolved by the third reviewer. A PRISMA flowchart, as shown in Figure 1, will be presented to demonstrate the results of screening and selection of studies.

### 2.11 Data extraction

Two reviewers will independently extract data from finally included studies using a standardized form. We will develop the data extraction form in MSExcel spreadsheet and it will be finalized after piloting on few included studies and thorough discussion among team members. From each articles, information will be extracted on year of publication, author name, study population and location, research design, details of the geriatric services provided, information on geriatric workforce, and measurements of outcome. A third reviewer will randomly check the data extraction process and resolve any disagreements between the reviewers.

### 2.12 Assessment of risk of bias

The risk of bias of the included studies will be assessed using the “risk of bias assessment tool for non-randomized studies” (RoBANS) tool.^[16]^ This tool mainly emphasizes on 5 major domains – selection bias, performance bias, detection bias, attrition bias, and reporting bias. The risk of bias will be assessed considering inclusion and exclusion criteria, sampling strategy, sample size, and assessment criteria of the outcomes. Two reviewers will independently assess the risk of bias and provide their judgements. Disagreements between the reviewers will be resolved by discussion and if deemed necessary third reviewer will be consulted to come into consensus. Additionally, we will examine the potential publication bias through funnel plot using the Review Manager software (Cochrane).

### 2.13 Data synthesis

Synthesis will be data driven. We are anticipating that mostly descriptive quantitative and qualitative studies will be included in the review, due to the nature of the outcome measure. Therefore, we will conduct a narrative synthesis of the interventions and the outcomes. The narrative synthesis will be thematically organized as the content and coverage of geriatric and palliative services provided in the health system, pattern of health workforce dedicated for the services, and association between the availability of different kind of health workforce and the available geriatric service coverage. Apart from the narrative synthesis, if quantitative studies are available that use same type of intervention and outcome measures, data will be pooled using a random-effect model meta-analysis. For the quantitative analysis, standardized mean differences will be presented for the continuous outcomes, while dichotomous outcomes will be reported analyzing the risk ratios. For each outcome, 95% confidence intervals and *P* values will be reported.

### 2.14 Analysis of subgroups or subsets

Subgroup analysis will be done to explore the different strategies taken for different cadres of healthcare providers such as doctors, nurses, and support staff. In addition, provided that enough data are found from regional context (South Asia, Africa), subgroup analysis will be done for those country settings.

### 2.15 Patient and public involvement

This systematic review and meta-analysis will analyze data from published articles on geriatric and palliative care, and there is no direct involvement of patients in the whole process.

### 2.16 Dissemination plans

The findings of the systematic review and meta-analysis will be presented to Government officials and relevant stakeholders including development partners, non-governmental organizations, academicians, and researchers through a series of interactive events in order to create linkage with other national-level actors in Bangladesh as well as the Global Health System and Human Resources Management. The aim will be to translate findings in a more visual and engaging format to reach a broad range of national, regional, and global stakeholders. Manuscripts will be submitted to peer-reviewed journals for publication. In addition, policy briefs will be prepared to communicate with the key policymakers.

## 3 Discussion

Advances in medical science coupled with increased income results in an elevated average of life expectancy even in resource-poor countries. This growing aged population has been lacking the adequate focus of much discussion in the health system context of LMICs. However, to ensure proper healthcare of these population segments, a coordinated health system response is crucial. Especially guaranteeing the availability of a properly trained and skilled health workforce is an absolute necessity for good geriatric and palliative healthcare. While there is a lack of specialist geriatricians in LMIC contexts, other health workforce strategies might be effective in building a proper health system response to this growing demand.

Therefore, this systematic review and meta-analysis will be conducted to identify the existing effective strategies taken to provide geriatric and palliative care, especially in LMICs. This study will synthesize evidences for service coverage and related the human resources allotted for geriatric healthcare. From LMIC's perspective, the increased life expectancy of people offers new challenge in terms of providing quality geriatric and palliative care. This is because the health system of these countries are yet to be full prepared to deal with the complex cases of geriatric and palliative issues. From this context, the findings of this study will help to generate evidence for developing policies and strategies, strengthen the health systems across to achieve universal health coverage.

### 3.1 Strengths and limitations of this study

This systematic review and meta-analysis is among the first few designed to synthesize evidence on the service coverage and effective health workforce allocation strategies for geriatric and palliative care in LMICs. The review process will be done using a rigorous search strategy adopting the state-of-the-art methodology – the Preferred Reporting Items for Systematic Reviews guideline. Considering the growing number of aged population in LMIC, the findings are expected to help policymakers identify the gaps and formulate new policies. Articles published only in English language will be included in the study, information from articles published in other languages will be missing.

## Acknowledgments

icddr,b is grateful to the Governments of Bangladesh, Canada, Sweden, and the UK for providing core/unrestricted support and commitment to icddr, b's research efforts.

## Author contributions

**Conceptualization:** Dipika Shankar Bhattacharyya, KM Saif-Ur-Rahman.

**Funding acquisition:** Dipika Shankar Bhattacharyya.

**Methodology:** Dipika Shankar Bhattacharyya, Goutam Kumar Dutta, Iffat Nowrin, KM Saif-Ur-Rahman.

**Project administration:** Dipika Shankar Bhattacharyya, Goutam Kumar Dutta.

**Software:** Goutam Kumar Dutta.

**Supervision:** Dipika Shankar Bhattacharyya, KM Saif-Ur-Rahman.

**Writing – original draft:** Dipika Shankar Bhattacharyya, Md. Hasibul Hossain, Goutam Kumar Dutta, Iffat Nowrin.

**Writing – review & editing:** Dipika Shankar Bhattacharyya, KM Saif-Ur-Rahman, Goutam Kumar Dutta, Iffat Nowrin Tuly.

## Supplementary Material

Supplemental Digital Content
